# The complete chloroplast genome of brown flatsedge, *Cyperus fuscus* (Cyperaceae)

**DOI:** 10.1080/23802359.2019.1660261

**Published:** 2019-09-03

**Authors:** Lan Ma, Wei Xu, Luhang Ma

**Affiliations:** School of Biological and Environmental Engineering, Xi’an University, Xi’an, China

**Keywords:** *Cyperus fuscus*, Illumina sequencing, MITObim, chloroplast genome

## Abstract

*Cyperus fuscus* known as brown flatsedge plays an important role in sand fixation and water purification. The first complete chloroplast genome sequences of pioneering plant *C. fuscus* were reported in this study. The complete chloroplast genome was 167,660 bp in length, containing a large single copy region (LSC) of 79,790 bp and a small single copy region (SSC) of 12,192 bp, which were separated by a pair of 37,839 bp inverted repeat regions (IRs). The chloroplast genome contains 135 genes, including 92 protein-coding genes, eight ribosomal RNA genes, and 35 transfer RNA genes. The overall GC content is 36.3%, while the corresponding values of the LSC, SSC, and IR region are 35.1%, 28.3%, and 38.5%, respectively. Further, the maximum-likelihood phylogenetic analysis showed a strong sister relationship with *Cyperus difformis* in Cyperaceae. Our findings provide a foundation for further study of chloroplast genome evolution and genetics information in *C. fuscus*.

*Cyperus fuscus* is a species of sedge, which known by the common name brown galingale or brown flatsedge. This plant is native to much of Europe, Asia, and North Africa from England, Portugal, and Morocco east to China and Thailand. *Cyperus fuscus* is aquatic and moist habitats, with high plant photosynthetic efficiency which could help it become a pioneering plant in poor environment (Obok 2012; Canavan et al. 2019). Also, *C. fuscus* was reported that play an important role in phytoremediation of heavy metal pollution in water and soils (Vara Prasad and de Oliveira Freitas 2003; Chang et al. 2009). To date, less genetic information on *C. fuscus* is reported. Here, we assembled and annotated the complete chloroplast genome of the *C. fuscus* using high-throughput sequencing technology, which will be helpful for further studies on the function of chloroplast genes and phylogenetic evolution.

The total genomic DNA was extracted from fresh leaves and high-throughput DNA sequencing was conducted on the Illumina HiSeq 2500 Sequencing System (Illumina, CA, USA) by Shanghai Genesky Biotechnologies Inc (Shanghai, China). The voucher specimen was collected in germplasm garden of Xi’an Botanical Garden of Shaanxi Province, Xi’an, China (34°12′32″N, 108°57′19″E) and stored at the laboratory of Institute of Botany of Shaanxi Province (LBJ1900293, XZY). Total 2.71 G raw reads were retrieved and trimmed by CLC Genomics Workbench v8.0 (CLC Bio, Aarhus, Denmark) and 1.05 G reads were aligned and ordered to the reference cp genome of *Hypolytrum nemorum* (GenBank: NC_036036.1) using NOVOPlasty (Dierckxsens et al. 2016). The chloroplast genome was annotated in GENEIOUS R11 (Biomatters Ltd., Auckland, New Zealand).

The chloroplast genome of *C. fuscus* is a circular DNA molecule with 167,660 bp in size (MK431855), containing a large single copy region (LSC) of 79,790 bp and a small single copy region (SSC) of 12,192 bp, which were separated by a pair of 37,839 bp inverted repeat regions (IRs). The total GC content of the chloroplast genome is 36.1%, while the corresponding values of the LSC, SSC, and IR region are 35.1%, 28.3%, and 38.5%, respectively. The chloroplast genome harbors 135 functional genes, including 92 protein-coding genes (PCGs), 35 tRNA genes, and eight rRNA genes. Among them, 55 are involved in photosynthesis, and 68 genes are involved in self replication. Moreover, among all the protein-coding genes, 19 genes contain one intron, whereas *atpA*, *chlp*, and *ycf3* harbour two introns.

The maximum-likelihood phylogenetic tree of *C. fuscus* was generated using those gene strings sequence by MEGA 6.0 (Tamura et al. 2013) with using 1000 bootstrap replicates. The 50 photosynthesis related PCGs sequences among 10 chloroplast genomes were aligned using MAFFT (Katoh et al. 2002) and then were connected as gene strings. The phylogenetic analysis (Figure 1) showed the position of *C. fuscus* was closely related to *Cyperus difformis* in Cyperaceae. Our findings provide valuable information on the genetic diversity research of *C. fuscus* and enrich the resources of chloroplast genomes in pioneering plants.

**Figure 1.**
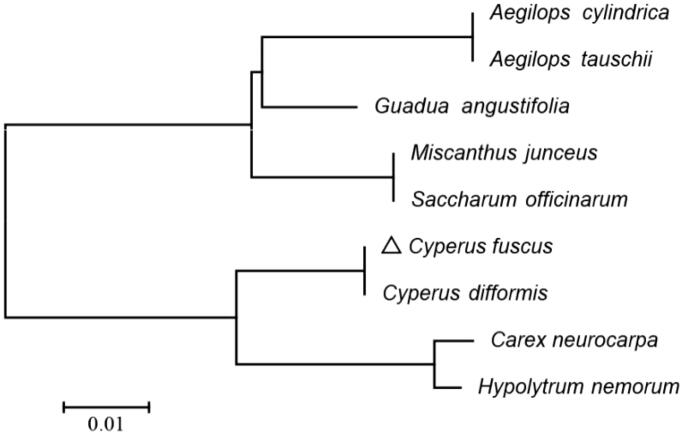
Phylogenetic of 10 species based on the maximum-likelihood analysis of the whole chloroplast genome sequences using 500 bootstrap replicates. The analyzed species and corresponding Genbank accession numbers are as follows: *Aegilops cylindrica* (NC_023096), *Aegilops tauschii* (NC_022133), *Carex neurocarpa* (KU238086), *Guadua angustifolia* (NC_029749), *Hypolytrum nemorum* (NC_036036), *Miscanthus junceus* (NC_035751), *Saccharum officinarum* (LN849913), and *Cyperus difformis* (MK423991).
